# Glutaric Aciduria Type I Missed by Newborn Screening: Report of Four Cases from Three Families

**DOI:** 10.3390/ijns7020032

**Published:** 2021-06-18

**Authors:** Johannes Spenger, Esther M. Maier, Katharina Wechselberger, Florian Bauder, Melanie Kocher, Wolfgang Sperl, Martin Preisel, Katharina A. Schiergens, Vassiliki Konstantopoulou, Wulf Röschinger, Johannes Häberle, Thomas Schmitt-Mechelke, Saskia B. Wortmann, Ralph Fingerhut

**Affiliations:** 1 Department of Pediatrics, Salzburger Landeskliniken (SALK) and Paracelsus Medical University (PMU), 5020 Salzburg, Austria; j.spenger@salk.at (J.S.); w.sperl@salk.at (W.S.); m.preisel@salk.at (M.P.); s.wortmann-hagemann@salk.at (S.B.W.); 2 Division of Metabolism, Dr. von Hauner Children’s Hospital, D-80337 Munich, Germany; esther.maier@med.uni-muenchen.de (E.M.M.); katharina.schiergens@med.uni-muenchen.de (K.A.S.); 3 Division of Neuropediatrics, Children’s Hospital Lucerne, CH-6004 Lucerne, Switzerland; katharina.wechselberger@luks.ch (K.W.); florian.bauder@luks.ch (F.B.); thomas.schmitt@luks.ch (T.S.-M.); 4 Kinderarztpraxis Arche, CH-3270 Aarberg, Switzerland; melanie.kocher@gmx.com; 5 Austrian Newborn Screening Program, Department of Pediatrics and Adolescent Medicine, Medical University of Vienna, 1090 Vienna, Austria; vassiliki.konstantopoulou@meduniwien.ac.at; 6 Division of Newborn Screening, Laboratory Becker & Colleagues, D-81671 Munich, Germany; w.roeschinger@labor-becker.de; 7 Division of Metabolism and Children’s Research Center, University Children’s Hospital Zurich, CH-8032 Zurich, Switzerland; Johannes.Haeberle@kispi.uzh.ch; 8 Swiss Newborn Screening Laboratory, University Children’s Hospital Zurich, CH-8032 Zurich, Switzerland

**Keywords:** glutaric aciduria type 1, newborn screening, glutaryl-carnitine, glutaric acid, 3-hydroxyglutaric acid, *GCDH* gene, missed cases

## Abstract

Glutaric aciduria type I (GA-1) is a rare autosomal-recessive disorder of the degradation of the amino acids lysine and tryptophan caused by mutations of the *GCDH* gene encoding glutaryl-CoA-dehydrogenase. Newborn screening (NBS) for this condition is based on elevated levels of glutarylcarnitine (C5DC) in dried blood spots (DBS). Here we report four cases from three families in whom a correctly performed NBS did not detect the condition. Glutarylcarnitine concentrations were either normal (slightly below) or slightly above the cut-off. Ratios to other acylcarnitines were also not persistently elevated. Therefore, three cases were defined as screen negative, and one case was defined as normal, after a normal control DBS sample. One patient was diagnosed after an acute encephalopathic crisis, and the other three patients had an insidious onset of the disease. GA-1 was genetically confirmed in all cases. Despite extensive efforts to increase sensitivity and specificity of NBS for GA-1, by adjusting cut-offs and introducing various ratios, the biological diversity still leads to false-negative NBS results for GA-1.

## 1. Introduction

Glutaric aciduria type I (GA-1) is a rare autosomal-recessive disorder of the degradation of the amino acids lysine and tryptophan caused by mutations of the *GCDH* gene encoding glutaryl-CoA-dehydrogenase (for review see [1]). Affected patients typically present in infancy or early childhood with progressive macrocephaly and an acute encephalopathic crisis, often caused by a catabolic state, e.g., a febrile viral illness. However, there are also patients who present with insidious onset dystonia, where clinical symptoms are less specific and show a slower progression. Accumulation of toxic metabolites may lead to irreversible damage of the basal ganglia and severe progressive dystonic cerebral palsy [2]. Patients with GA-1 can also be biochemically subdivided into two groups. One are the high excretors, where marker metabolites in urine are always elevated, with urinary glutaric acid > 100 mmol/mol creatinine. The second group are the low excretors, where marker metabolites are normal, or not detectable with the standard methods for the analysis of organic acids in urine, with urinary glutaric acid < 100 mmol/mol creatinine [3]. Guidelines for screening, diagnosis, and treatment were recently published [4], and have proven success in improving the outcomes of patients [5,6]. An early diagnosis and prevention of metabolic deterioration is the key for this, and therefore newborn screening (NBS) for this condition has been done in Bavaria since 1999, in Austria since 2004, and in Switzerland since November 2014, based on elevated levels of glutaryl-carnitine (C5DC) in dried blood spots (DBS). Despite extensive efforts to increase sensitivity and specificity of NBS for GA-1 by adjusting cut-offs and introducing various ratios, the biological diversity still leads to false-negative NBS results for GA-1 [5,7,8,9,10].

## 2. Case Reports

### 2.1. Case 1

**Figure 1:**
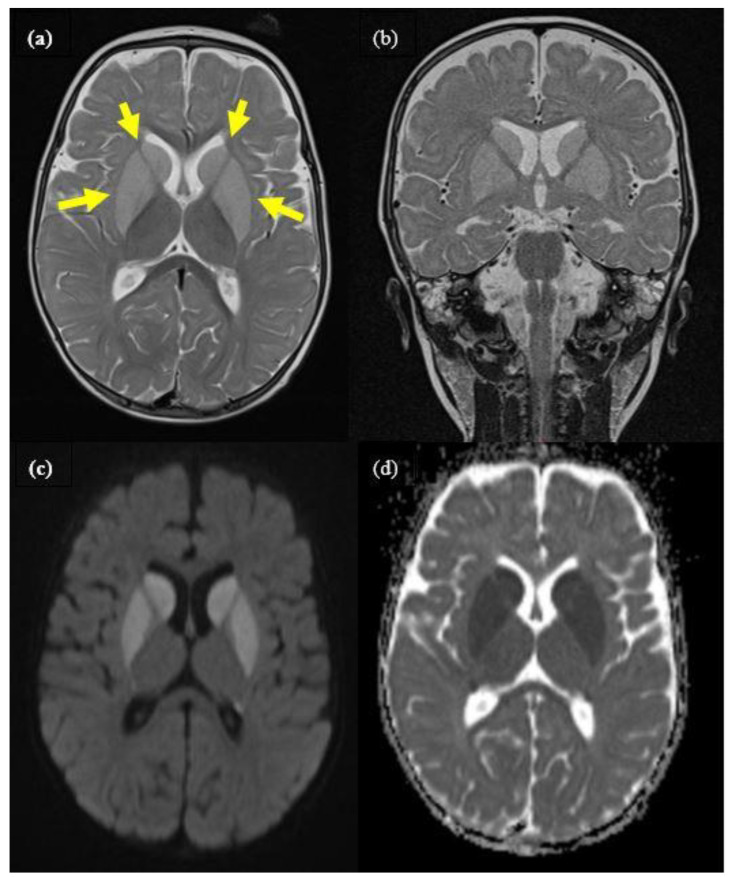
Cranial MRI of patient 1 during encephalopathic crisis at the age of 9 months, showing distinct bilateral signal alteration of caudate, putamen, and pallidum (yellow arrows). (**a**): axial T2, (**b**): coronar T2, (**c**): axial diffusion weighted (DWI), (**d**): ADC-map imaging.

Patient 1 was born at term after an uneventful pregnancy. The boy was the second child of healthy, non-consanguineous Swiss parents. NBS was performed correctly with unremarkable results. His previous development and head circumference were normal. At the age of 9 months, he presented to the emergency department with fever, vomiting, diarrhea, and mild dehydration. His body weight was 9.45 kg (62nd percentile, 0.31 Z-score), length was 73 cm (39th percentile, −0.28 Z-Score), and head circumference was 46 cm (63rd percentile, 0.34 Z-Score). His older brother also was suffering from gastroenteritis. He was admitted due to several short clonic seizures with eye deviation. Serum electrolytes and glucose were normal, and rotavirus antigen was positive in feces. The initial diagnosis was convulsions associated with rotavirus gastroenteritis, and treatment with low-dose carbamazepine was started. His condition worsened the next day with the development of an acute encephalopathic crisis, including muscular hypotonia, sleepiness, and repeated dystonic posturing with paroxysmal eye movements. Cranial magnetic resonance imaging (MRI) showed bilateral signal alterations of the basal ganglia affecting caudate, putamen, and pallidum (Figure 1), suggestive of GA-1. Carnitine supplementation and a protein-restricted diet were started according to current guidelines [4] and the family received an emergency plan. Metabolic testing showed elevation of urinary glutaric acid and 3-hydroxyglutaric acid (table). Diagnosis of GA-1 was genetically confirmed, showing a compound heterozygous *GCDH* genotype with pathogenic variants c.722G>T/p.Gly241Val (mutation not previously described) and c.1169G>C/p.Gly390Ala. Subsequently, the boy developed dystonic cerebral palsy and a global developmental delay. At the age of 23 months, he was still not able to sit, but was not dependent on tube feeding and had preserved good social contact. At his last visit at the age of 5 years, he showed dyskinetic cerebral palsy (GMFCS Level 5) but good social contact, and he is still not dependent on tube feeding.

### 2.2. Case 2

Patient 2 was the first child of healthy non-consanguineous Moroccan parents. Pregnancy and birth were unremarkable, as was NBS. He had a surgical correction of an obstructive mega-ureter detected after pyelonephritis with consecutive malfunction of the right kidney.

**Figure 2:**
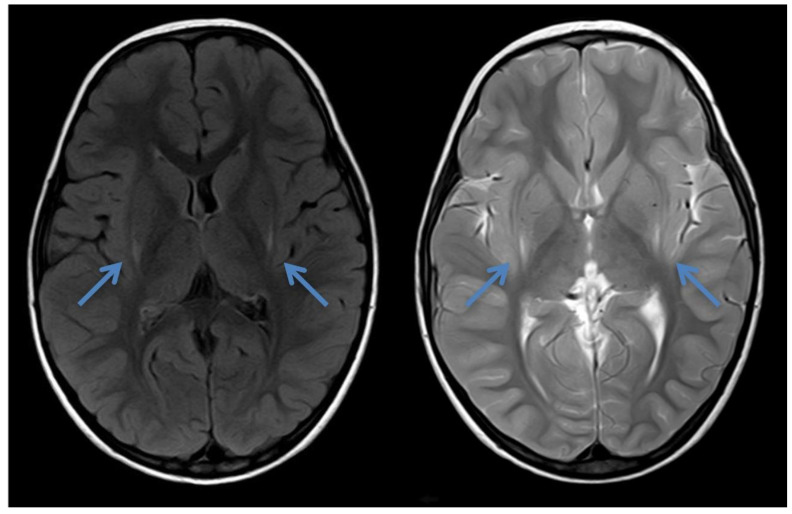
Cranial MRI of patient 2 at the age of 2 years and 8 months showing laterodorsal increased signal intensity bilaterally around the putamen (blue arrows) in axial FLAIR and T2—the typical insidious onset pattern of GA-1.

At the age of 2 years and 4 months, he was first referred to the neuropediatrician because of non-progressive ataxia and developmental delay. A cranial MRI at the age of 2 years and 8 months showed laterodorsal mild increased signal intensity around the putamen in T2 and FLAIR (Figure 2). Plasma amino acids and urinary organic acids were unremarkable, and a serum acylcarnitine profile was not performed. At that time, the MRI findings were not interpreted as a typical insidious onset pattern of GA-1. The parents did not follow up with further outpatient appointments, and he was brought to medical attention again at the age of 10 years when his sister (patient 3) was evaluated.

At that time, the parents reported that he never had any deteriorations upon fasting or intercurrent illnesses and that the movement disorder had improved significantly over time. They further reported that he was attending a regular school but still has problems with the German language (mother tongue: Arabic), and there were behavioral issues in his contact with peers. The physiotherapist reported an improvement in balance, coordination, and fine motor skills (e.g., writing without tremor) but a remaining tendency to fall compared with tests at the age of 3 years (compared with age-matched controls). Physical examination at the age of 10 years showed age-adequate height (33rd percentile), weight (23rd percentile), and head circumference (66th percentile). Walking and running were unremarkable, but he frequently showed short nodding of the head or dystonic movements of one hand. Tightrope walking was slightly instable and the finger-to-nose test was unremarkable. At the age of 10 years, genetic testing revealed previously described compound heterozygous variants in *GCDH* (p.Arg257Gln, p.Met405Val) [11,12], confirming the diagnosis of GA-1. The patient was frequently advised to follow a low-protein, almost vegetarian diet. It is questionable if the recommended diet is followed, and the same holds for carnitine supplementation. Now aged 14 years, the patient is in regular school without learning difficulties and shows a discrete dystonic movement disorder without subjective limitations in daily activities.

### 2.3. Case 3

**Figure 3:**
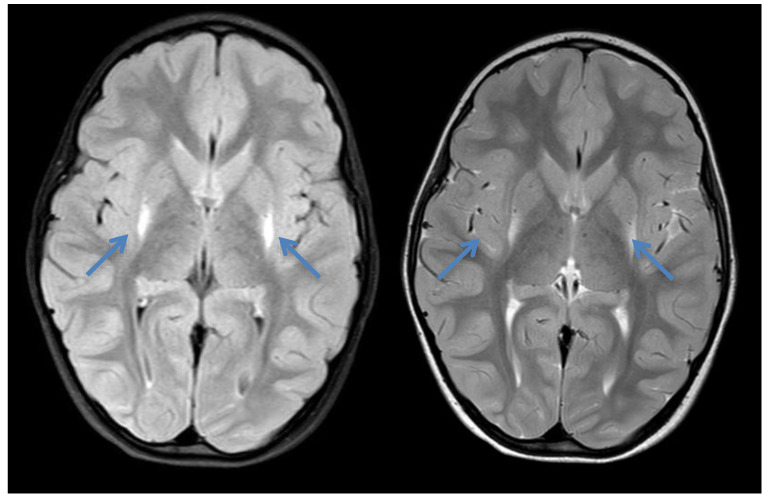
Cranial MRI of patient 3 at the age of 4 years showing increased signal intensity bilaterally around the putamen (blue arrows) in axial T1 and T2—the typical insidious onset pattern of GA-1.

Patient 3 is the younger sister of patient 2 and the third child in the family. One older sister is healthy. Pregnancy, birth, and development were unremarkable. The NBS revealed values within normal ranges. At the age of 18 months, she was referred to the neuropediatrician because of delayed motor development, but follow-up appointments were not attended. At the age of 4 years, she was again brought to medical attention because of dystonic movements. Cranial MRI at that age showed increased signal intensity bilaterally around the putamen in axial T1 and T2—the typical insidious onset pattern of GA-1 (Figure 3). Urinary organic acids and serum acylcarnitines showed the typical profiles for GA-1. Parents reported no deteriorations upon fasting or intermittent illnesses. After the diagnosis was made (compound heterozygous variants in *GCDH*: p.Arg257Gln and p.Met405Val), she was admitted during an infection with poor oral intake, and a clear deterioration of the movement disorder was observed. She rapidly regained previous skills and showed intermittent titubation and an instable gait, with a tendency to fall upon intermittent short dystonic posturing of the legs. An MRI control was not performed due to the lack of clinical consequence and the necessity of anesthesia. Despite several attempts and even admission to our ward for dietary advice, it remains questionable if the recommended diet is followed and the prescribed carnitine is taken. Compared with her brother (patient 2), patient 3, currently 8 years of age, shows a distinct dystonic movement disorder and learning difficulties with special requirements and support in school.

### 2.4. Case 4

Patient 4 is a girl, the first child of healthy, non-consanguineous German parents (ethnicity was not reported). She was born after an uneventful pregnancy via Cesarean section due to pelvic presentation. Her postnatal adaptation was unremarkable with normal APGAR scores (10/10), a birth weight of 3335 g (42nd percentile), length of 52 cm (58th percentile), and head circumference of 36.5 cm (93rd percentile). NBS at the age of 47 h revealed a slightly elevated concentration of C5DC and two of three ratios slightly elevated. On repeated analysis in DBS at the age of 8 days, a normal concentration of C5DC was found.

During her first months of life, she showed normal development. Retardation of motor development was first noticed at the age of 10 months by the parents. She did not achieve unassisted sitting before the age of 11 months.

At the age of 16 months, she was seen by a neuropediatrician. She presented with muscular hypotonia, instable posture, and mild dystonia. At that time, she was able to stand when assisted but unable to walk. Metabolic and genetic diagnostics were initiated. Lumbar puncture revealed normal concentrations of neurotransmitters in the CSF. An MRI of the brain was not performed. A genetic panel analysis compiling 98 genes associated with ataxia was not informative. Analysis of organic acids in the urine at the age of 21 months showed an elevated concentration of 3-hydroxyglutaric acid. Concentrations of C5DC in DBS and plasma were slightly elevated. Mutation analysis of the *GCDH* gene revealed the previously described pathogenic variant c.769C>T/p.Arg257Trp and the novel variant c.510G>C/p.Lys170Asn. Pathogenic relevance of the latter was confirmed by determination of a low residual GCDH activity of 7% in leukocytes.

Notably, she suffered from a highly febrile illness for several days at the age of 1 year, as well as a norovirus infection at the age of 20 months. During both illnesses, no neurological deterioration was observed.

As of the last follow-up at 26 months, she is on a lysine-restricted diet (80 mg/kg) and receives oral carnitine (100 mg/kg) and physiotherapy, and she shows continuous developmental progress. Her posture is still instable. However, she now walks when assisted. Her fine motor skills are delayed by 7 months, and her gross motor skills are delayed by 14 months. Cognitive functions are unaffected. The clinical phenotype can be classified as “insidious onset”.

## 3. Results

Glutarylcarnitine (C5DC) in DBS was normal in the NBS cards of patients 1, 2, and 3 (Table 1, Table 2 and Table 3) and was slightly elevated in patient 4 (Table 4), with normal ratios and normal values in a second sample from day 8. It was also normal at the time of diagnosis and during follow-up. Even after carnitine supplementation, C5DC remained normal. The concentrations of the marker metabolites glutaric acid and 3-hydroxyglutaric acid in urine were only slightly elevated. The diagnosis was confirmed by mutation analysis of the *GCDH* gene. Patients 2 and 3 were compound heterozygous for p.Arg257Gln and p.Met405Val, patient 1 was compound heterozygous for p.Gly241Val and p.Gly390Ala, and patient 4 was compound heterozygous for p.Arg257Trp and the novel variant p.Lys170Asn.

## 4. Discussion

On the basis of excretion of glutaric acid and 3-hydroxyglutaric acid, all 4 patients belong to the group of low excretors [3]. This is supported by the genetic variants that were detected. All four cases are compound heterozygous and have one severe mutation (0% residual activity or missense mutation) on one allele. Three have a mild mutation (4–25% residual activity) on the second allele, and only patient 1 has a previously unknown mutation, for which the residual activity is not yet known. Patients 2 and 3 are compound heterozygous for a previously described severe mutation (p.Arg257Gln) with 0% residual activity [11] and a milder mutation (p.Met405Val) with 4–25% residual activity that is more prevalent in African patients [12]. Patient 4 is also compound heterozygous for one previously described mutation (p.Arg257Trp) [11], which is predicted to be a missense mutation [13], and a previously undescribed mutation (p.Lys170Asn). With a detected residual activity of 7%, the mutation p.Lys170Asn is considered rather mild. Patient 1 is also compound heterozygous for one previously described mutation (p.Gly390Ala), which is also predicted to be a missense mutation [13], and a previously unknown mutation (p.Gly241Val). However, in view of the mild biochemical phenotype, this should also be considered a mild mutation. It must be emphasized that in this context, mild means that the mutation is most likely related to a biochemically low excretor status. However, high excretors and low excretors generally show the same clinical outcomes. Patients with the low excretor subtype typically present either with an acute encephalopathic crisis or with insidious onset dystonia. 3-Hydroxyglutaric acid seems to be the best marker, as it was at least slightly elevated in all instances but one. However, in the literature [3], normal levels have also been reported. C5DC and several ratios are not reliable markers for the detection of GA-1. Even in the catabolic newborn period (36–96 h of life), when blood for the routine NBS is taken, C5DC was only slightly elevated in patient 4, borderline normal in patient 1, and completely normal in patients 2 and 3. Interestingly, the sample of patient 4 was taken at 47 h of life, while the other samples were taken on day 4. In all DBSs that were taken later for confirmation or follow-up, C5DC was only borderline once. Only some of the ratios were elevated, but unfortunately, none of the five ratios that were used seem to be reliable primary markers. As a result of the missed cases, the cut-off for the primary marker C5DC was deliberately lowered to 0.35 μmol/L, accepting an increased number of false-positive cases that will need a complete diagnostic workup. In Austria, cut-offs have also been adjusted, and in Germany, all newborns with positive NBS results will be directly referred to a metabolic center for a complete diagnostic workup.

## 5. Conclusions

Normal C5DC in NBS cannot completely exclude GA-1. In addition, a normalized C5DC after a borderline elevated screening result cannot exclude GA-1. The low excretors can have normal C5DC even under carnitine supplementation. In case of a positive first NBS result or a clinical suspicion of GA-1 even if the child had a normal NBS result, a complete workup including molecular genetic testing is necessary. This is also recommended in the guidelines of the German Society for Pediatrics and Adolescent Medicine (DGKJ) [14]. In addition, as long as there are no additional markers or marker ratios found that give a positive result even in anabolic situations, GA-1 cannot be excluded if the timely NBS sample (approx. 36–96 h of life) is not taken or lost during transportation.

## Figures and Tables

**Table 1:** Laboratory results of patient 1. Free carnitine and glutaryl-carnitine are in µmol/L. DBS result from day 4 was from the NBS laboratory; all other DBS results were from the clinical chemistry laboratory. The cut-offs in both laboratories are different; organic acids are in mmol/mol creatinine; n.d. = not done.

Age	4 d	Reference Range	9 m	10 m	13 m	15 m	18 m	24 m	Reference Range
**DBS**									
Free carnitine	26	7–55	26	45	51	38	35	46	11–44
Glutarylcarnitine	0.5	<0.53	0.03	0.04	0.04	0.06	0.01	0.04	<0.06
**Urine**									
Glutaric acid	n.d.		26	<10	<10	n.d.	n.d.	<10	0–20
3-hydroxyglutaric acid	n.d.		22	19	23	n.d.	n.d.	17	0–10

**Table 2:** Laboratory results of patient 2. Free carnitine and glutaryl-carnitine are in µmol/L; organic acids are in mmol/mol creatinine. n.r. = not reported; n.d. = not done.

Age	4 d	2 y 8 m	10 y	11 y	Reference Range
**DBS**					
Free carnitine	n.r.	n.d	n.r.	37.5	7.0–70.0
Glutarylcarnitine	0.34	n.d.	0.27	0.30	<0.2
C5DC/C2	0.01	n.d.	n.r.	n.r.	<0.01
C5DC/C4	n.r.	n.d.	1.29	5.09	0.02–0.32
C5DC/C8	2.01	n.d.	9.25	6.73	<8
C5DC/C12	n.r.	n.d.	11.2	0.84	0.05–0.83
C5DC/C16	0.13	n.d.	n.r.	n.r.	<0.19
**Urine**					
Glutaric acid		normal	normal	n.d.	0–20
3-hydroxyglutaric acid		normal	13	n.d.	0–10

**Table 3:** Laboratory results of patient 3. Free carnitine and glutaryl-carnitine are in µmol/L; organic acids are in mmol/mol creatinine. n.r. = not reported; n.d. = not done.

Age	4 d	4 y	5 y	Reference Range
**DBS**				
Free carnitine	-	n.r.	42.5	7.0–70.0
Glutarylcarnitine	0.19	normal	0.16	<0.2
C5DC/C2	0.01	n.r.	n.r.	<0.01
C5DC/C4	0.76	n.r.	0.54	0.02–0.32
C5DC/C8	3.17	2.21	4.12	<8
C5DC/C12	n.r	n.r.	2.91	0.05–0.83
C5DC/C16	0.05	n.r.	n.r.	<0.19
**Urine**				
Glutaric acid	n.d.	normal	n.d.	0–20
3-hydroxyglutaric acid	n.d.	14.2	n.d.	0–10

**Table 4:** Laboratory results of patient 4. Free carnitine and glutaryl-carnitine are in µmol/L; organic acids are in mmol/mol creatinine. n.r. = not reported; n.d. = not done.

Age	47 h	8 d	Reference Range	21 m	Reference Range
**DBS**					
Free carnitine	16.2	13.1	6–47	n.r.	n.r
Glutarylcarnitine	0.78	0.38	<0.45	0.12 ^#^	<0.09
C5DC/C4	2.39	1.52	<1.87	n.r.	n.r.
C5DC/C8	5.97	7.5	<5.60	n.r.	n.r.
C5DC/C12	1.6	2.27	<1.92	n.r.	n.r.
**Plasma**					
Glutarylcarnitine	n.d.	n.d.		0.35	<0.23
**Urine**					
Glutaric acid	n.d.	n.d.	0–20	n.r.	0–20
3-hydroxyglutaric acid	n.d.	n.d.	-	8.66	0.17–1.17

^#^ The sample taken at 21 months was measured in a different laboratory with a different reference range.
