# Correction: Spenger et al. Glutaric Aciduria Type I Missed by Newborn Screening: Report of Four Cases from Three Families. *Int. J. Neonatal Screen*. 2021, *7*, 32

**DOI:** 10.3390/ijns8010002

**Published:** 2021-12-31

**Authors:** Johannes Spenger, Esther M. Maier, Katharina Wechselberger, Florian Bauder, Melanie Kocher, Wolfgang Sperl, Martin Preisel, Katharina A. Schiergens, Vassiliki Konstantopoulou, Wulf Röschinger, Johannes Häberle, Thomas Schmitt-Mechelke, Saskia B. Wortmann, Ralph Fingerhut

**Affiliations:** 1 Department of Pediatrics, Salzburger Landeskliniken (SALK) and Paracelsus Medical University (PMU), 5020 Salzburg, Austria; j.spenger@salk.at (J.S.); w.sperl@salk.at (W.S.); m.preisel@salk.at (M.P.); s.wortmann-hagemann@salk.at (S.B.W.); 2 Division of Metabolism, Dr. von Hauner Children’s Hospital, D-80337 Munich, Germany; esther.maier@med.uni-muenchen.de (E.M.M.); katharina.schiergens@med.uni-muenchen.de (K.A.S.); 3 Division of Neuropediatrics, Children’s Hospital Lucerne, CH-6004 Lucerne, Switzerland; katharina.wechselberger@luks.ch (K.W.); florian.bauder@luks.ch (F.B.); thomas.schmitt@luks.ch (T.S.-M.); 4 Kinderarztpraxis Arche, CH-3270 Aarberg, Switzerland; melanie.kocher@gmx.com; 5 Austrian Newborn Screening Program, Department of Pediatrics and Adolescent Medicine, Medical University of Vienna, 1090 Vienna, Austria; vassiliki.konstantopoulou@meduniwien.ac.at; 6 Division of Newborn Screening, Laboratory Becker & Colleagues, D-81671 Munich, Germany; w.roeschinger@labor-becker.de; 7 Division of Metabolism and Children’s Research Center, University Children’s Hospital Zurich, CH-8032 Zurich, Switzerland; Johannes.Haeberle@kispi.uzh.ch; 8 Swiss Newborn Screening Laboratory, University Children’s Hospital Zurich, CH-8032 Zurich, Switzerland

There was an error in the original publication [1]. In patient 2 and 3, a mutation (p.Arg257Glu) is mistakenly documented. Instead, the mutation is p.Arg257Gln. A correction has been made to Section 2. Case Reports, 2.2. Case 2, Paragraph 3; Section 2. Case Reports, 2.3. Case 3, Paragraph 1; Section 3. Results, Paragraph 1; Section 4. Discussion, Paragraph 1:

Section 2. Case Reports, 2.2. Case 2, Paragraph 3: At the age of 10 years, genetic testing revealed previously described compound heterozygous variants in *GCDH* (p.Arg257Gln, p.Met405Val) [11,12], confirming the diagnosis of GA-1.

Section 2. Case Reports, 2.3. Case 3, Paragraph 1: After the diagnosis was made (compound heterozygous variants in *GCDH*: p.Arg257Gln and p.Met405Val), she was admitted during an infection with poor oral intake, and a clear deterioration of the movement disorder was observed.

Section 3. Results, Paragraph 1: Patients 2 and 3 were compound heterozygous for p.Arg257Gln and p.Met405Val, patient 1 was compound heterozygous for p.Gly241Val and p.Gly390Ala, and patient 4 was compound heterozygous for p.Arg257Trp and the novel variant p.Lys170Asn.

Section 4. Discussion, Paragraph 1: Patients 2 and 3 are compound heterozygous for a previously described severe mutation (p.Arg257Gln) with 0% residual activity [11] and a milder mutation (p.Met405Val) with 4–25% residual activity that is more prevalent in African patients [12].

The authors apologize for any inconvenience caused and state that the scientific conclusions are unaffected. The original publication has also been updated.
